# Revisiting values in evaluation: exploring the role of values in shaping evaluation practices and their influences on decision-making within English higher education providers

**DOI:** 10.1007/s10734-024-01335-6

**Published:** 2024-10-28

**Authors:** Catherine Kelly

**Affiliations:** Massachusetts Institute of Technology, 77 Massachusetts Ave, Cambridge, MA 02139 USA

**Keywords:** Values, New Public Management, Evaluation use, Higher education, Social equity, Organisational behaviour

## Abstract

Theoretical and empirical contributions to research on evaluation have advanced our understanding of how values influence evaluation practice. Yet rather than understand how values shape evaluation and its use, research on the evaluation of widening participation (WP) programmes delivered by English higher education (HE) providers has focused on methodological deficits. Rather, this study explores the complexity of how national policy, organisational imperatives and the individual values of staff responsible for WP within HE providers influence how evaluation is practised and used to inform decision-making. The results of semi-structured interviews with 17 staff members spanning the organisational hierarchy of three diverse English HE providers highlight conflicts between staff values, job roles and responsibilities and espoused organisational values, and how they can influence symbolic and legitimising evaluation practices. Alternatively, at the individual level staff values support the process and instrumental use of evaluation to inform programme improvements. The findings identify implications for how HE providers can shape their evaluation systems, and how staff choose to enact evaluation within their programme areas.

## Introduction

Arguing that evaluation is not a value-neutral activity, evaluation scholars continue to theorise about the role of values in shaping evaluation practices (House, 2015; Schwandt & Gates, 2021) and have conducted research exploring how values influence evaluation practice (LaVelle et al., 2022; Teasdale et al., 2023). Despite this advancement in our understanding of the role of values in evaluation, research about how values influence evaluation practice is still relatively rare compared to other areas, such as evaluation practice around use and stakeholder participation (Coryn et al., 2017). In addition, Raimondo (2018) has also identified gaps in the empirical evaluation literature focused on how actors within organisations respond to having to evaluate their policies, programmes and projects through organisational systems of evaluation. This is surprising because systems of evaluation are increasing, especially within English higher education (HE) providers delivering on their widening participation (WP) agenda who, under principles of New Public Management, are embedding systems of evaluation into their organisational practices and procedures (Harrison et al., 2018; OfS, 2019). These systems are supported by the Director for Fair Access and Participation, who has recommended the sector “evaluate, evaluate, evaluate” (Blake, 2022), and the Office for Students (OfS), which states in their regulatory guidance that “evaluation should be undertaken by a provider on an ongoing basis” (OfS, 2023, p. 17).

Alongside the growth of routinised evaluation practices in English HE providers, research on the evaluation of WP programmes has tended to critique methodological decisions, such as the lack of evidence-based gold standard methodologies (Gorard & Smith, 2006; Younger et al., 2018), or why HE providers should adopt more complexity informed and theory-based methodologies (Clements & Short, 2020; Harrison & Waller, 2017). On the other hand, research exploring values within WP has centred on the practice of developing and delivering WP programmes (e.g. Ingram & Gamsu, 2022; Stevenson et al., 2010), including how they should be evaluated (Hayton & Bengry-Howell, 2016). For example, Hayton and Bengry-Howell (2016) describe how the Network for Evaluation and Researching University Participation Interventions (NERUPI) framework can be used to enable a “more strategic approach to planning, delivering, and evaluating interventions and programmes of activity” (p.51). This framework is rooted in praxis, recognising the transformative potential of utilising practitioner knowledge and insight (Burke, 2018). Thus, praxis encompasses the important role of practitioner values and beliefs within its methodology. This study adds value to this literature by explicitly exploring the complexity of how values influence evaluation and its use to inform programmatic and strategy decision-making. This is important because values are known to shape how evaluation is implemented (Teasdale, 2021) and how likely evaluation will be used to inform decisions (Greene, 1987; Patton, 1997). For example, being responsive to stakeholder needs and producing valid and credible evaluation findings require understanding stakeholder values (Greene, 1990). Consequently, there are calls for more critical reflection on how values influence evaluation practices (Hall et al., 2012; Schwandt & Gates, 2021).

This study addresses gaps in the HE, WP, and evaluation literature by examining the complex interplay between national policy, organisational imperatives and staff values that influence WP-based evaluation practices and the influence of evaluation on decision-making within three diverse English HE Providers. First, literature examining the context of WP within English HE is provided, including how evaluation in WP has become systematised within national policy and regulation through principles of New Public Management. Then, a conceptual framework explores how job roles, responsibilities and values influence how staff in English HE providers practise and use evaluation to inform decision-making. Finally, the implications of this study are explored through a thematic analysis of interview data with 17 staff members spanning the organisational hierarchy of 3 diverse English HE Providers.

## A background to widening participation policy and evaluation in English HE

The publication of the Dearing Report in 1997 solidified WP within the lexicon of HE policy (Thompson, 2017), emerging when England faced an increasingly decentralised and marketised HE system (Bessant et al., 2015). WP projects and programmes focus on supporting students from disadvantaged and underrepresented groups to access and succeed within HE (Moore et al., 2013). Yet, diverse values and objectives underpin WP policy and activities (Burke, 2016). On the one hand, WP policy helps institutions to recruit more students to increase their income (McCaig, 2011). It also provides an opportunity for transformative change towards greater social justice (Burke, 2016) and to achieve equity of opportunity (e.g. through contextualised admissions) over equality of opportunity (e.g. by academic merit alone) (Boliver et al., 2022). The dominant view, however, is that WP supports a social mobility agenda, focused on changing individual behaviours and attitudes so that students enter and succeed within higher education, reaping economic returns (Burke, 2016). Thus, the design, implementation and delivery of WP policy and activity are driven by a complex array of values and objectives.

Through the New Labour government (1997–2010) and the national WP agenda since 1997, there has been an increasing emphasis on producing evidence-based policy (Crockford, 2020), which has solidified the importance of evaluation for WP policy. For example, evaluation helped to inform the discontinuation of the flagship Aimhigher programme (Doyle & Griffin, 2012). Since 2006/7, HE providers have been required to produce monitoring returns outlining their WP targets (Crockford, 2020). Presently, the setting of targets for WP is formalised through the development of access and participation plans, regulated through the OfS. These plans enable institutions to operationalise national-level policy into the institutional form, describing their organisational imperatives to deliver and evaluate their WP programmes to meet their targets (Rainford, 2019).

## Implications of the WP evaluation system

Following principles of New Public Management where performance is quantified and monitored as a way to motivate individuals or groups to act in line with an organisation’s goals (Van der Kolk, 2022a), in WP, there is an expectation that HE providers will set and continuously review their targets for the students they are supporting to progress and succeed within HE (OfS, 2022). Evaluation systems developed through principles of New Public Management are known to have different effects on performance. In HE, performance management delivered with fairness, clarity and communication can positively affect employee commitment and performance (Decramer et al., 2012), whereas incentive-oriented and high-pressure performance management can increase job-related stress (Van der Kolk, 2022b). In an evaluation context, employees with lower motivation to do their work can experience lower evaluation capacity, and the opposite is true when employees have higher motivation driven by internal factors (Sen et al., 2023). Thus, how an evaluation system becomes embedded within a policy area can influence the policy enactment process.

The rise of performance management has corresponded with an increase in evaluation systems, where evaluation becomes routinised within organisational practices (Leeuw & Furubo, 2008). Raimondo and Leeuw (2021) argue that evaluation systems can fall prey to bureaucratic capture when evaluation “loses its instrumental function of informing decisions and speaking truth to power” (p. 145). This happens when evaluations are overly positive rather than scrutinising bureaucratic processes to benefit the public they intend to serve (Raimondo & Leeuw, 2021). Similarly, Van der Kolk (2022a) describes the hidden costs of performance management such as when easy indicators are selected to measure targets to give the illusion that they are being met, or focusing only on short-term targets at the expense of potential long-term consequences. In WP, this is known as ‘deadweight’, where students likely to progress to HE without any intervention are targeted for support (Harrison, 2012). Deadweight provides the illusion that programmes are effective, which helps to feed the bureaucracy instead of holding it to account.

Likewise, Dahler-Larsen (2012) refers to the constitutive effects of systematised evaluation practices. Constitutive effects occur when evaluation systems define and shape organisational practices including the programmes being evaluated. Deadweight can be a constitutive effect of evaluation systems. In WP, constitutive effects are also present when HE providers design activities so they can implement a randomised control trial to evaluate them, rather than defining the evaluation method around the activity (Clements, 2023). In these cases, evaluation can be used as a performative tool to create an illusion of rigour, rather than supporting the critical reflection of WP practice that can help instigate transformative change (Clements, 2023; Greene, 2015). These practices can produce a symbolic and legitimising effect of evaluation (Raimondo, 2018).

To address many of these challenges, an alternative position is provided which recommends a deliberative and democratic evaluation approach which actively represents and involves stakeholders in the evaluation process, acknowledging their values and valuing processes (Dahler-Larsen, 2023; Greene, 2015; House & Howe, 2000). Therefore, as this paper argues, it is important to build on our understanding of the complex interplay of values at an organisational level, directed through the evaluation system, and at the individual level, influencing how staff within HE providers enact their WP policy and evaluation. Building this understanding can support the critical reflection of HE providers and employees on how they shape evaluation systems and enact their evaluation practices. This is so they can steer evaluation to advance their organisational imperatives to widen participation to increase social equity and social mobility and avoid implementing evaluation practices that might impede these goals (e.g. by incentivising deadweight).

## Conceptual framework: the role of values in evaluation systems

### Personal values

According to Schwartz (1992), value systems are developed by individuals and groups. Values comprise six main features: they represent beliefs, refer to desirable goals, transcend actions and situations, serve as standards and criteria, are ordered by importance, and guide action (Schwartz, 2012). An individual can hold multiple values, including amongst others, professional, social, cultural, epistemic and political values (Hassall et al., 2020; Teasdale et al., 2023). Whilst other values are likely to inform evaluation practice and decision-making, this study focuses explicitly on the epistemic and social equity values at the individual and organisational levels.

### Epistemic values

Epistemic values signify a belief in the most appropriate approach to generating knowledge, including what counts as methodological rigour (Hassall et al., 2020). Schwandt argues that “method debates are generally proxies for deeper differences surrounding what evaluation should be examining” (Schwandt, 2015). Thus, epistemic values can be informed by other values, such as social and professional values (Teasdale et al., 2023). Evaluation systems embed evaluation practices into the organisational routines and procedures, following a distinct epistemological perspective (Leeuw & Furubo, 2008). In WP, the espoused epistemic values of the organisation are influenced by the OfS, who require HE providers to evaluate their WP provision, adopting defined standards of evaluation evidence (OfS, 2019). Influenced by evidence-based policy focused on understanding ‘what works’, the standards of evidence situate evaluation activity into three types, the first is evidence underpinned by narrative or theory of change, the second includes pre- and post-evaluation designs that cannot determine causality, and the third type includes randomised control trials and quasi-experimental evaluation designs (OfS, 2019).

Some practitioners and organisations, including the OfS, advocate using experimental methodologies including randomised control trials and quasi-experimental designs whenever feasible (OfS, 2019; Younger et al., 2018). However, this epistemological perspective can differ at the individual level. For example, other practitioners opt for theory-based and interpretive models for evaluation that focus on examining the lived experiences of communities and students (Austen, 2022; Clements & Short, 2020; Formby et al., 2020). Others support using praxis-based frameworks for evaluation that centre on practitioner expertise and knowledge (Hayton & Bengry-Howell, 2016). Of course, these choices are not always mutually exclusive. Rather, they highlight the variety of values underscoring how practitioners may choose to implement evaluation of their activities and the potential conflicts between the epistemic values advanced by the evaluation system and the personal epistemic values of practitioners (Schwandt & Gates, 2021). They also illustrate the various levels of capacity and expertise required to implement different evaluation methods (TASO, 2023).

### Social equity values

**Fig. 1:**
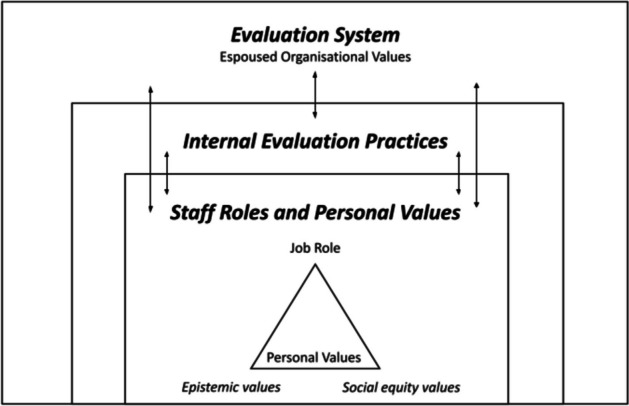
Conceptual framework illustrating the influence of organisational values, personal values and job roles on internal evaluation practices

Teasdale et al., (2023) define values underpinning evaluators' perception of equity and justice as ‘values related to social betterment’. In their study, values related to social betterment address evaluators' beliefs about the broader purpose of evaluation in society. In the conceptual framework in Fig. 1, these values are listed as ‘social equity values’. This is because WP is rooted in a variety of social values including increasing social mobility, and social justice.

Differences in social equity values can affect how staff choose to evaluate their programmes (Teasdale et al., 2023). For example, evaluation in WP based on a need to achieve social justice tends to centre the need for the system to change to accommodate students from underrepresented and disadvantaged backgrounds (Burke, 2016). Alternatively, evaluation based on a belief in social mobility tends to put the focus of change on the individual student, such as to ‘raise their aspirations’ (O’Sullivan et al., 2019; Spohrer et al., 2018; Wisker & Masika, 2017).

### Job roles and responsibilities

Staff with responsibility for evaluation have different job roles (Harrison et al., 2018) which may also affect how they choose to achieve their values through enacting WP policy and its evaluation. Given that an individual will modify their attitudes and behaviours to fulfil their values (Arieli et al., 2020), job roles can affect how staff choose to live out their values (Hemingway & Maclagan, 2004. For example, staff operating within strategic roles are more likely to use knowledge symbolically, compared with staff working operationally to develop and deliver policy (Boswell, 2008). Within WP, there is a known distinction between how managers view the purpose of WP and how it should be enacted compared with other staff members or the wider organisation (Greenbank, 2006; Stevenson et al., 2010).

## Methods

This study examines how values influence WP-based evaluation practices and how evaluation influences decision-making within three diverse English HE Providers. To achieve this aim, the study follows a constructivist and pragmatic approach to knowledge generation, recognising that humans create knowledge in different and often changing contexts and that the knowledge we produce changes as we interact with the world (McWilliams, 2016). Constructivism has been linked to Deweyan pragmatism based on Dewey’s belief in language and communication in the construction of meaning, particularly meaning constructed through participation and interactions with others (Garrison, 1995). Considering the exploratory nature of the study and the constructivist approach applied, interviews were chosen as the single research method for data collection because they are most useful when the purpose of the research is to understand individual perspectives and experiences (Roulston & Choi, 2018).

During the interviews, respondents were invited to reflect on their role in evaluation within their department and their motivations and values driving their involvement in evaluation, how they perceive other staff to be involved in the evaluation of their activities, and to reflect on their perceptions of their HE provider’s approach to evaluating their WP programmes. They were then asked to explain how they collect and analyse data and any priorities internally or externally that affect their evaluation practice. The interview protocol ended by asking respondents how evaluation has affected their WP practice and organisational strategy decision-making.

### Sampling strategy and rationale

Rather than focusing on the point of view of evaluators, it was important to include staff members spanning the organisational hierarchy of three substantively different HE providers, a Russell Group University, a Post-1992 University and a Further Education College (FEC). This is because HE providers have different amounts of resources and different targets for their WP programmes which means their organisational structures and approaches to enacting WP policy and its evaluation are likely to differ (McCaig, 2016; Rainford, 2017). Summarised in Table 1, a total of 17 staff representing the organisational hierarchy of each HE provider were interviewed between 60 and 90 min for this study.

**Table 1:** Summary of interview participants by HEP, job role and department

HE provider	Job role	Area/department
Russell Group University	Evaluator	WP
	WP practitioner	
	Administrative assistant	
	Head of WP	
	Graduate intern	
	Learning manager	Library
	Faculty-based WP practitioner 1	Faculty
	Faculty-based WP practitioner 2	Faculty
Post-1992 University	Senior WP practitioner	Widening access
	WP facilitator	
	Head of WP	
	Evaluation manager	Policy unit
	Senior manager	
	Senior leader	
FE College	Recruitment & admissions manager	Recruitment & admissions
	Vice principal	Student experience
	HE quality manager	

### Methods of analysis

The thematic analysis began with the transcription of the interview data, which was recorded using a Dictaphone. Unfocused transcription was used because the main goal of the research was to generate themes and patterns across the data (King & Horrocks, 2010). First, codes were developed based on my familiarity with the conceptual framework, the WP and evaluation literature, and my experiences working in WP and evaluation. I read each excerpt and noted concepts that articulated participants’ experiences. I reflected on the semantic meanings expressed by participants and the potential latent meanings (Terry et al., 2017). For example, a WP Facilitator said “You’ll write it (an evaluation report), you’ll spend lots of time on it, but the internal recommendations, once you’ve sent it to the school… you never revisit the report or the evaluations, the evaluation sometimes gets shredded anyway”. On the face of it, the WP Facilitator described a form of non-use. Internally, the evaluation reports are not reviewed after they have been written. This quote was initially coded as ‘non-use’. More latent meanings within the data suggest how evaluation can be used to legitimise the HEP and WP department. An external report is provided to schools, but internally, nothing else happens. The HE provider has evaluated their work and then shredded the reports. This excerpt was also coded as ‘legitimisation’.

Then, after generating the first list of codes, patterns were identified. According to Terry et al. (2017), it is important for themes to address the research aims, and to be meaningful considering the other themes that are developed. Each theme developed from the codes in this study describes how values affect evaluation practice and how evaluation influences practice and strategy decision-making within the sampled English HE providers. For example, non-use and legitimisation represent behaviours related to reporting evaluation findings. Alongside other codes that also represented the dissemination of evaluation reports, I developed an initial theme to describe how the influence of evaluation differs depending on where evaluation is reported within the organisation. The first iteration of the theme became “evaluation influence differs depending on the level at which it is being reported to within the organisational hierarchy”. After reflecting on the data and the initial themes, it became apparent that they were topic summaries rather than central organising concepts akin to Braun and Clarke’s approach to thematic analysis (Braun et al., 2022). This theme was changed to a central organising concept, ‘making tweaks’, which centres on participants’ experiences of using evaluation findings symbolically or only making very small-scale changes to their programmes because they already perceive them to be impactful.

### Findings

#### Theme 1: methodological (mis)alignments

By attempting to satisfy their values and the perceived values of senior staff members and their organisation, staff embed multiple methods for evaluation that they think can meet the needs of individual colleagues. In some cases, operational staff can perceive senior staff to hold different epistemic values, such as placing more value on performance monitoring “Sometimes senior leaders just want simple stats, they want simple answers to simple questions that don’t necessarily answer the problems we’re setting out to solve”—Senior WP Practitioner, Post-1992. These epistemic values intersect with their job roles. In the case of the Post-1992 University, the Senior WP Practitioner perceived some senior leaders to value methods that do not align with their own beliefs about what type of data is needed to “get into the nitty gritty of what parts of the activity are working”.

But these perceptions are not always true, the Senior Policy Manager at the same Post-1992 University stated they are interested in “understanding the lived experience and wanting to gain that appreciation and understanding to find out more about what creates unequal outcomes and inequity”. These perceptions can change how data is collected and what types of data are shared across the organisation to fulfil procedural requirements and staff members' job roles. In both the Post-1992 and Russell Group University, data showing how many students have engaged with WP programmes are being fed up to senior leaders for reporting, whilst more formative insights are kept for use by the practitioners themselves—“in terms of the actual success of the event, I wasn’t feeding it back to anyone”—Faculty-based WP Officer, Russell Group.

Conflicts between epistemic values and job responsibilities are partly based on the extent to which practitioners in the study feel they need to follow guidance from the OfS. For example, the Head of WP at the Post-1992 University shared that providing causal assessments of their activity on student progression into university is “beyond difficult because what they’re trying to do is apply a methodology that’s used in a controlled situation in a lab or for a very small timeframe”. Rather, many staff interviewed stated that they valued using more creative and qualitative evaluation methods and incorporated them into the delivery of their programmes.

Conflicts between the epistemic values of individuals and the espoused epistemic values of the organisation result in WP teams using participant surveys in most of their programmes. The data gathered from these surveys tends to satisfy their institution's perceived notion of impact which is target-driven through the access and participation plan. The WP Facilitator at the Post-1992 University explained that their surveys are “a range of questions—they rate the day and each session on a scale of 1–5”. The same approach is evident within the Russell Group University with a focus on what they call “asking students core questions”. Due to resource constraints and different staffing structures, evaluation processes differ slightly within the FEC. Although they do collect data using surveys and focus groups, participants from the FEC explain how, when evaluating their programmes, they are more likely to use data from their monitoring information system including application and conversion rate figures (i.e. the number of students they have worked with who ended up applying and entering their institution).

Although interviewees have their epistemic values, data is shared across the organisation depending on how practitioners perceive the epistemic values of their managers. This means that, rather than implementing a unified evaluation process, bits of evaluative data are collected and shared with different audiences. Rather than alleviating tensions, this process adds to the conflicts staff feel when they must collect and share data which they do not believe to be of value for their job role.

#### Theme 2: external validation

The creation of the OfS, with its targeted approach to WP delivered through access and participation plans, and their standards of evidence, has influenced the perceptions and views of staff towards their evaluation practice. In some instances, the influence of the OfS complements the social equity values of managers and senior leaders operating strategically because “they’re clearer about their expectations and put support in place to do it”—Evaluation and Evidence Manager, Post-1992. Moreover, the Senior Leader in the Post-1992 University believes the approach from the OfS has led to a more concise focus on equality and equity, which has been led from the top through the Vice-Chancellor who supports their goals.

But these external pressures also conflict with the social equity values of administrators, practitioners and evaluators and their ability to fulfil their more operational job roles. For example, the WP Practitioner at the Russell Group University expressed they “hate it (evaluation) because there’s no support for it and it’s massively under-resourced”. In the Post-1992 University, the Senior WP Practitioner struggles with the capacity and resources to evaluate *why* certain activities may support or prevent them from being met. The alignment of their social equity values for WP and perceived organisational values/policy objectives creates distinct methodological tensions within their evaluation practice.

To some managers and senior leaders, including from the Post-1992 University, the change enforced by the OfS has meant that they have had to think about their WP work from a new strategic perspective, such as being able to better explain the student journey through an outcome rather than output-oriented evaluation. However, this is not the case for staff from the FEC which has fewer resources to deliver their WP agenda. The HE Quality Manager from the College expressed conflict between meeting targets and their social equity values over providing WP students the opportunity to study at their institution. From their perspective, targets for student success (e.g. students passing their degree with a grade of 2:2, 2:1 or higher) may prevent their institution from providing places for disadvantaged students who may achieve a lower grade than their targets, but for whom HE may be beneficial. Overall, these tensions mean that there is a split between types of evaluation occurring for different audiences which are not always in sync as a whole process. There is a form of monitoring to measure whether targets have been met and a separate formative evaluation to help practitioners understand the student experience.

#### Theme 3: making tweaks

Across each HE provider, practitioners discussed how they rely on their own experiences and observations throughout the delivery of a WP programme to inform whether their programmes need changing. The WP Facilitator at the Post-1992 University described their process, stating.

My main priority is to assess the learning outcomes based on the evaluation that’s collected and normally the presence in the room as well, so how well the learners have engaged.

The faculty-based WP Practitioner at the Russell Group University follows a similar process and often makes concrete changes to their activity based on their observations. Similarly, the Graduate Intern at the Russell Group University received student feedback about how best to support students with their personal statements, which led to improvements in the information and advice provided to students.

In some cases, the focus is on making small changes to already established programmes because, as the Graduate Intern at the Russell Group University explains “I think the work we do going forward is tweaking an already quite successful programme or learning as much as we can to improve that programme specifically but not introduce new ideas as much”. The Senior WP Practitioner at the Post-1992 University shared a similar experience using evaluation findings from their end-of-year report, limited to ‘tweaking individual activities’. For the Administrative Assistant at the Russell Group University, the small changes to activities tend to be logistical, in line with the focus of their job role.

In some instances, larger conceptual and practical changes to WP programmes or events are based on a combination of experiences through the evaluation process, findings, and the priorities of the sector. The Head of WP at the Post-1992 University explained how they changed a programme intended to increase student attainment by providing revision support to a programme aimed at improving students’ meta-cognition after “looking around at what other universities were doing” which benefited the team because they “were having real issues with putting on the programme”. Interestingly, from the perspective of the Senior WP Practitioner at the same institution, the changes to the revision programme were made based on feedback from students and teachers. This aligns with their job functioning at a more operational level.

The further up the organisational hierarchy, the influence of evaluation appeared to change from small-scale behavioural changes to a more symbolic use of evaluation facilitated through report sharing, because other departments will want information “if they’re writing a bid for something, an award submission, or want to put something in the university magazine”—Head of WP, Post-1992. The Russell Group University follows similar practices with their annual report, described by their Evaluator as being used to highlight the successes of their work. In this case, the University has an external narrative of its work alongside an internal narrative, which is more informal and focused on both the positive and negative findings from their evaluations. The Evaluator explained these discussions are “our way of sharing best practices, sharing what hasn’t gone well, what could be adapted to other programmes”. Similarly, staff at the FEC report using evaluation to celebrate the impact they are having on students. Reflecting on the way evaluation makes them feel, the Recruitment and Admissions Manager stated “Evaluation is a good way to capture everything you’ve done and celebrate the positive differences made”.

Through a need to demonstrate programme impact, there is a performative nature to WP-based evaluation practice, which has sometimes led to the symbolic use of evaluation. In this case, symbolic use occurs because the university and individual staff members tend to hold preconceptions of impact before evaluating their work. The Head of WP explained that “our evaluation, a lot of what we do and put in place is how we’re measuring impact and how we’re measuring success”. The faculty-based WP Officer at the Russell Group University echoed the same sentiment “(evaluation is) collecting the data to show that what we’re doing has an impact, a positive impact”. The WP Practitioner at the Russell Group University explained the importance of evaluation because “we need to demonstrate all of our interventions are leading to young people of colour going into HE”. With these expectations, practitioners feel the need to prove their reason for existence within their institution. When evaluation is reported internally through team meetings more negative findings will be discussed, and programmes are often adapted for improvement. When evaluation is reported externally, programmes are positioned as impactful and transformative.

#### Theme 4: persuasion is part of the game

When operational staff feel they are delivering their social equity values, evaluation becomes a means of persuasion and justification. For example, the Learning Manager at the Russell Group University explains how evaluation can be used to persuade funders about why a new initiative should be funded or given the go-ahead,

If you let me do this new thing, promise, I can show you that in 18 months we will be doing ‘this’, based on my understanding of the data, looking at the trend and some kind of experienced logical best guess.

Evaluation is also used to persuade different types of staff members, including academics, about why an existing programme or activity is useful. The Senior WP Practitioner at the Post-1992 University used a theory of change completed through the evaluation process as a persuasive tool to explain to faculty members why their activities are not random but “fit as part of a multi-intervention project”. In other situations, evaluation findings are used to persuade staff members about why it is important to uphold certain values and attitudes towards students who may be identified as needing to receive WP programmes. This is particularly prevalent in the Russell Group University, as explained by the Head of WP,

In terms of where we are trying to make that case to influence things, having that clear evidence base is important, and also using it to challenge the misconceptions about WP students as well…particularly around contextual admissions and students coming in on lower offers.

Some staff use different methods of evaluation to reinforce the decisions they make about the effectiveness or relevance of certain programmes. The Student Recruitment Manager at the FEC explained how qualitative and anecdotal data “serves to reinforce the (quantitative) data”. Likewise, describing how they are conflicted between being impartial to the data and using it to reinforce their reasoning behind why the programme exists, The WP Practitioner at the Russell Group University explained “I will sometimes just focus on the quotes that reinforce what I’ve been, that the research has been saying and reinforce the reasons why it needs to be this way”.

#### Theme 5: strategic and enlightening

According to some practitioners, evaluation within HE providers has helped to shift some attitudes about WP. In the FEC, the Vice Principal describes this process as staff developing an understanding of equality and equity, stating.

I think it’s also been quite surprising to academic staff who have maybe not noticed the difference, you know this attitude of we treat every student the same so if they don’t achieve it’s just coincidence that they also happen to be mature students, I think the evaluation has helped them to realise that there is.

For many interviewees, the act of producing a theory of change has contributed to greater levels of evaluative thinking. The Evaluation and Evidence Manager at the Post-1992 University explained how they are “constantly thinking about the ultimate goal before thinking about what I’m doing about it”. This view was echoed by the faculty-based WP Practitioner from the Russell Group University who acknowledged becoming more goal-oriented in their work. Similarly, the Senior WP Practitioner at the Post-1992 University found the challenge of completing theories of change “has helped me work out exactly how to go about the project in the best way”. In other instances, evaluation has helped to facilitate critical thinking around the wider systemic basis for WP policy within a HE provider. This includes providing a deeper focus on the structuring of activities rather than the effects of activities on students, as the Senior Leader at the Post-1992 University shared.

The evaluation findings showed us that the tools we were using to support student success weren’t necessarily effective because of poor student attendance... This demonstrated to me that there wasn’t an issue with the students it was in understanding why students don’t attend, and that fed into the development of the policy.

## Discussion and conclusion

### The WP evaluation system influences evaluation practice

WP-related evaluation practice is influenced by systems of New Public Management and performance management (McCaig, 2011). This is reflected in the target-driven approach to regulation through institutional access and participation plans (Crockford, 2020). As evaluation has become an intrinsic feature of the WP policy enactment process in HE providers, staff members responsible for enacting this policy by delivering programmes and evaluating them are influenced by the need to meet and report on their targets (Van der Kolk, 2022b).

### Conflicts between job roles, responsibilities and personal values influence evaluation practice

Some staff members within operational job roles have epistemic values that differ from their senior leadership or the wider organisation (Stevenson et al., 2010), which can cause conflicts and tensions about what data should be collected and why. In these cases, formative data about the student experience is kept for use by practitioners but does not reach further up the hierarchy to support more strategic decision-making. This can contribute to the bureaucratic capture of evaluation within HE providers because data collection and analysis is more likely to support their views that their programmes are ‘impactful’, rather than providing opportunities to critically reflect on their practices (Clements, 2023). Alternatively, other aspects of the evaluation process (as opposed to evaluation findings), including theory of change development, provide opportunities for practitioners to reflect on their programme development.

In some cases, evaluation is used as a performative tool to achieve programmatic aims driven by the social equity values and job roles and responsibilities of staff. This follows the findings from Arieli et al. (2020) that individuals will modify their attitudes and behaviours to fulfil their personal values. In this case, staff acknowledged using evaluation to persuade management that their ideas for new programmes will be impactful and using qualitative data to reinforce their targeting findings. This reflects a form of indicatorism where staff are using the evaluation system to their advantage so they can implement their practice and achieve their social equity values and job roles and responsibilities (Van der Kolk, 2022a).

### Complex interplay between the evaluation system, values and job roles and responsibilities affect evaluation influence

Similarly, in cases where staff feel restricted by the evaluation system and the requirements set by the OfS, they are less likely to implement evaluation methods that they value more highly, like creative and qualitative methods that can provide details about the lived experiences of their target students (Clements et al., 2021). This can contribute to the need for staff to use evaluation to their advantage. In the process, the information fed to senior leaders can have more symbolic uses as staff include the most positive evaluation findings in annual reports and the wider university communications (Boswell, 2008), often excluding negative findings.

Despite instances of symbolic and non-uses of evaluation, this study illustrates how evaluation that is directly responsive to stakeholder needs (i.e. operational staff members), mainly formative evaluation and theory of change processes, can support staff to improve and modify existing programmes (Greene, 1990). In some cases, evaluation methods and processes that do not affect the targeting data shared with management through the evaluation system can influence changes made to activities and programmes. Still, the performative nature of the WP evaluation system, prompting symbolic uses of evaluation, suggests that more transformational changes to WP programmes and strategy may be less likely to occur (Boswell, 2008; Clements, 2023).

This has implications for HE providers and their ability to deliver on their WP agendas to advance social mobility. It is evident in this study that many staff within HE providers have epistemological perspectives that can differ from the espoused values of their organisation as advanced by the regulators of HE. Therefore, along with evaluation scholars including Greene (2015), House and Howe (2000) and Dahler-Larsen (2023), this study takes the view that there should be more deliberation about evaluation systems and more critical reflection and acknowledgement of the role of the personal values of staff shaping decisions in evaluation processes.

## Limitations and implications for future research: revisiting values in evaluation

As an explorative qualitative study employing thematic analysis, these findings are not intended to be generalisable—however, the limitations of this study signal directions for future research on the topic. First, the sample of participants is more heavily weighted to the Russell Group and Post-1992 University. Whilst reflective of their larger number of dedicated resources for WP, the study could have been improved by including a larger and more diverse group of participants. Furthermore, the study has only examined specific types of personal values that may influence evaluation practice including epistemic and social equity values. Future studies may benefit from exploring a wider range of personal values, including distinguishing between the personal values of managers and operational staff members and how they influence staff behaviours concerning their organisational evaluation practices.

Despite the limitations, the findings of this study align with findings from other fields including evaluation (Teasdale et al., 2023), organisational behaviour (Arieli et al., 2020), and public management (Van der Kolk, 2022b). Whilst presented here in the context of English HE and the policy of WP, the findings may be useful for informing evaluation policy development and enactment across other HE providers outside of England. Broadly speaking, the implications of ignoring the valuing process within evaluation can lead to an increase in bureaucratic capture. Therefore, it is imperative that the sector critically assess the values that are driving the development of evaluation systems (Schwandt & Gates, 2021). Regulators of HE and senior leaders within HE providers responsible for their WP agenda should explore how they could adopt a more deliberative and democratic approach to developing WP evaluation policies and standards. This would begin by actively reflecting on and explicitly identifying the values and valuing processes informing the development of evaluation systems and implementation of evaluation practices (Greene, 2015). Doing so can advance evaluation for learning and action and support organisations to deliver their agendas for widening participation.

## Data Availability

Data supporting this study can be accessed through the UK Data Service ReShare repository.
